# Olive Cultivars Susceptible or Tolerant to *Xylella fastidiosa* Subsp. *pauca* Exhibit Mid-Term Different Metabolomes upon Natural Infection or a Curative Treatment

**DOI:** 10.3390/plants10040772

**Published:** 2021-04-15

**Authors:** Chiara Roberta Girelli, Laura Del Coco, Federica Angilè, Marco Scortichini, Francesco Paolo Fanizzi

**Affiliations:** 1 Department of Biological and Environmental Sciences and Technologies, University of Salento, Prov.le Lecce-Monteroni, 73100 Lecce, Italy; chiara.girelli@unisalento.it (C.R.G.); laura.delcoco@unisalento.it (L.D.C.); federica.angile@unisalento.it (F.A.); 2 Council for Research in Agriculture and Economics-Research Centre for Olive, Fruit and Citrus Crops, Via di Fioranello 52, I-00134 Roma, Italy; marco.scortichini@crea.gov.it

**Keywords:** *Xylella fastidiosa*, ^1^H-NMR spectroscopy, metabolomics, susceptible cultivar, tolerant cultivar

## Abstract

*Xylella fastidiosa* subsp. *pauca,* is a bacterial phytopathogen associated with the “olive quick decline syndrome” (OQDS) causing severe economic losses to olive groves in Salento area (Apulia, Italy). In a previous work, we analyzed by ^1^H-NMR the metabolic pattern of naturally infected Ogliarola salentina and Cellina di Nardò susceptible cultivars untreated and treated with a zinc-copper citric acid biocomplex and we observed the treatment related variation of the disease biomarker quinic acid. In this study, we focused also on the Leccino cultivar, known to exhibit tolerance to the disease progression. The ^1^H-NMR-based metabolomic approach was applied with the aim to characterize the overall metabolism of tolerant Leccino in comparison with the susceptible cultivars Ogliarola salentina and Cellina di Nardò under periodic mid-term treatment. In particular, we studied the leaf extract molecular patterns of naturally infected trees untreated and treated with the biocomplex. The metabolic Leccino profiles were analyzed for the first time and compared with those exhibited by the susceptible Cellina di Nardò and Ogliarola salentina cultivars. The study highlighted a specificity in the metabolic response of the tolerant Leccino compared to susceptible cultivars. These differences provide useful information to describe the defensive mechanisms underlying the change of metabolites as a response to the infection, and the occurrence of different levels of disease, season and treatment effects for olive cultivars.

## 1. Introduction

In Italy, the cultivation of olive (*Olea europea* subsp. *europea* var. e*uropea*) is mainly concentrated in the central-southern areas of the country, and Apulia region accounts for the largest occurrence of the crop [1], this representing one of the most relevant incomes for the local economy. In Salento area, olive is cultivated on about 30% of the total hectares present in Apulia [2], but the occurrence of the “olive quick decline syndrome” (OQDS) is threatening the survival of the crop [3]. In this area, Ogliarola salentina and Cellina di Nardò are the main cultivars traditionally present since ancient times [4] and nowadays used for high nutritional value oil production [5]. Unfortunately, both cultivars are very susceptible to the OQDS [3]. The disease causes severe leaf, twig and branch wilting followed, in many cases, by the plant death. *Xylella fastidiosa* subsp. *pauca* is the quarantine bacterium frequently associated with the syndrome and is retained as the causal agent of the disease [6]. Besides the Ogliarola salentina and Cellina di Nardò, another olive cultivar introduced in Salento is Leccino. This latter is considered an ancient autochthonous cultivar of Tuscany (central Italy), cultivated in many countries for its adaptability to different agricultural systems [7]. Leccino is more tolerant to *X. fastidiosa* subsp. *pauca* than the local Salento cultivars by showing both less concentration of the bacterium cells within the xylem tissue of the infected plants and transcriptome profiles assessed upregulation of genes related to the plant defense mechanisms [8].

Some studies have been already performed trying to find out features that can possibly explain the tolerance of the cultivar towards the bacterium. An increase in the total lignin content within the branch and a differential expression of lignification related-genes has been found in Leccino trees infected by *X. fastidiosa* subsp. *pauca*, suggesting a critical role of lignin for the cultivar tolerance [9]. Upon infection, Leccino also yields, with respect to the susceptible cultivar Cellina di Nardò, less xylem cavitation and occlusions as well as an intense mechanism of xylem refilling through aggregation of starch grains [10]. Moreover, a diverse and quite stable endophytic microbiota has been found in Leccino trees either in healthy condition or when infected by the bacterium [11]. The metabolomic approach based on nuclear magnetic resonance (NMR) spectroscopy in combination with chemometrics allows the simultaneous detection for a wide range of structurally different metabolites, providing useful information for specific sample discrimination as in plant studies [12,13]. Beside the simultaneous detection of various components, this powerful fingerprinting technique also offers other advantages with respect to other conventional analytical methods (GC, HPLC, GC/HPLC-MS). These include high reproducibility for comparable data with high statistical confidence level and minimal requirements for sample amount and preprocessing [14]. Several studies proved the NMR-based screening techniques as a successful tool for biomarker detection, food quality control and/or origin discrimination [15,16,17,18]. By using an NMR based approach, specific involvement of some xylematic polyphenols and carbohydrates in the *X. fastidiosa* subsp. *pauca* interaction with the susceptible cultivars Ogliarola salentina and Cellina di Nardò were observed and described in our previous work [16]. In particular, we analyzed for the first time the effect of Dentamet^®^ on *X. fastidiosa* infected olive trees. We focused on the preliminary short time effect on the metabolic profiles of Cellina and Ogliarola cultivars, which showed a different incidence and severity of disease before of the treatments [16]. Increased quinic acid, as a disease marker for the bacterium infection, was also confirmed by ^1^H-NMR analysis of xylematic extracts [15,19]. The metabolomic analysis of leaf extracts also allowed to ascertain that, upon a curative treatment with a zinc-copper-citric acid compound (Dentamet^®^) [20], infected cultivars undergo early reprogramming of some metabolic pathways leading to a consistent increase of malic acid in Ogliarola salentina and of γ–aminobutyric acid (GABA) in Cellina di Nardò [15]. Both cultivars also showed an increase of oleuropein, ligstroside and phenolic compounds, besides quinic acid, upon natural infection [15]. In the present work, we studied by ^1^H-NMR metabolic profiling and multivariate statistical analyses the xylematic extracts of the *X. fastidiosa* subsp. *pauca*-tolerant cultivar Leccino in comparison with the susceptible cultivars Ogliarola salentina and Cellina di Nardò following a mid-term period (after several performed cycles) of Dentamet^®^ spray treatment. The metabolic profiles evaluation focused on a comparison, in a specific time span, during a multiyear field treatment schedule, of untreated and Dentamet^®^ treated *Xylella* susceptible (Ogliarola salentina and Cellina di Nardò) and tolerant (Leccino) trees, naturally infected by the bacterium.

## 2. Results and Discussion

### 2.1. Selection of Leccino, Ogliarola Salentina and Cellina Di Nardò Metabolic Profiles in a Mid-Term Treatment Study

Real-time PCR analyses confirmed the occurrence of *X. fastidiosa* subsp. *pauca* in the selected Galatone trees [21] and its absence in the Grottaglie ones. At Galatone, the visual assessment of twig wilting progression across the seasons revealed a significant decrease in the Dentamet^®^-treated trees when compared with control trees [21]. In order to clearly evaluate and possibly understand the effects of mid-term Dentamet^®^ treatment on different *X. fastidiosa* subsp. *pauca*-infected cultivars, a specific timespan was chosen during a multiyear on field usage schedule. This latter lasted three years (2017–2019) and consisted in spraying the product on the tree canopy of both susceptible (Ogliarola salentina and Cellina di Nardò) and tolerant (Leccino) trees once a month for 6 consecutive months (from April to September included) during each year. No treatment was supplied over the remaining months. The aim of the study was to observe, in the different studied cultivars, the treatment effectiveness over a specific cycle (after several performed cycles). The experimental design (Table 1) also allowed evaluation of possible memory effects due to previous treatments persisting after suspension periods (6 months). Therefore, metabolomic analyses were performed on leaf samples extracts from infected trees in two specific samplings over the last treatment year (2019). The first leaf sampling was arranged in early March 2019, just before the starting of the third year treatment, after 6 months from the last of the two previous treatment cycles suspension. The second sampling (early October) was carried out at the end of the six months of the third year treatment. Samples from untreated controls trees were also collected during the two sampling periods from surviving infected plants of the same cultivars, growing in the bordering groves.

### 2.2. General Effects of Sampling Period and Treatment on Leaf Extracts Metabolic Profiles for Naturally Infected Leccino, Cellina Di Nardò and Ogliarola Salentina

**Figure 1:**
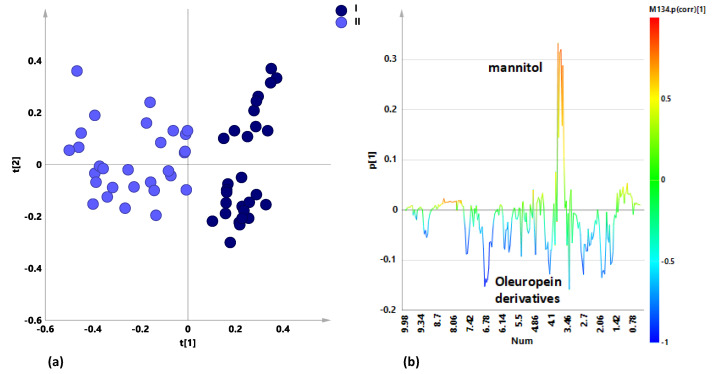
(**a**) Principal components analysis (PCA) t1/t2 scores plot for naturally infected Ogliarola salentina, Cellina di Nardò and Leccino leaf samples. Symbols were colored according to the sampling periods. (**b**) Line plot for the model, indicating the ^1^H-NMR chemical shifts of the signals, characteristic of specific metabolites, discriminating the classes along t[1] and colored according to the correlation-scaled loading p(corr).

The preliminary unsupervised analysis (PCA) performed on the whole dataset constituted by naturally infected Leccino, Ogliarola salentina and Cellina di Nardò leaf extracts ^1^H-NMR spectral data showed a clear “natural” grouping of the leaf extracts according to the sampling period (Figure 1a).

**Figure 2:**
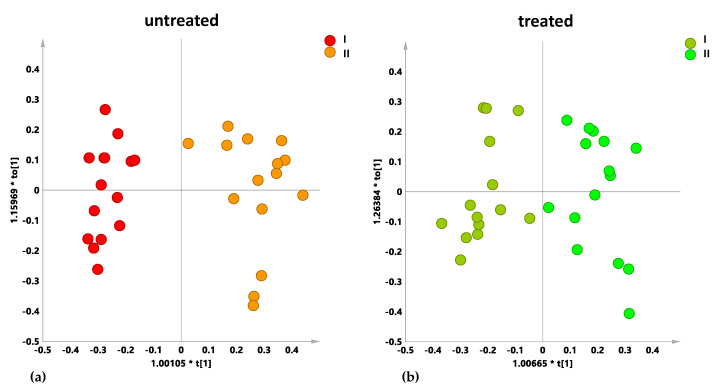
Orthogonal partial least square–discriminant analysis (OPLS-DA) t[1]/t[2] scores plot for untreated (**a**) and treated (**b**) naturally infected Leccino, Cellina di Nardò and Ogliarola salentina leaf samples comparing the I and II sampling periods.

The model is satisfactory described by three components that gave 0.69 and 0.518 for R2X and Q2, respectively. Visual inspection of the t1/t2 scores plot showed that infected leaf samples from both untreated and treated trees grouped according to the sampling periods along the t1 component. The related loading plot (Figure 1b) clearly indicated a major contribution of mannitol (3.66, 3.78 ppm) and oleuropein derivatives (7.58; 6.86 ppm) metabolites for the differentiation along the first component. Looking at the two separate PCA models obtained for untreated and treated samples, respectively, a different degree of separation along the first component was observed (Figure S1a,b). Although more pronounced in untreated with respect to treated samples the separation along the t1 was found as essentially related to the mannitol contribution (Figure S1b,c). This suggested that, irrespective of treatment, season dependence for this metabolite appeared enhanced in *X. fastidiosa* subsp. *pauca*-infected cultivars with respect to previous observations for healthy trees [22]. The important role of sugar metabolism in *X. fastidiosa* subsp. *pauca*-infected cultivars was already pointed out in previous metabolomics analyses [15,16] and recently focused on specifically increased mannitol content [23]. According to our observation the mannitol content appeared decreased in the early fall (October) with respect to March sampling. Interestingly this natural seasonal decrease of mannitol was also observed for the treated samples although to a lesser extent (minor contribution to the t1 component in the specific PCA). This was confirmed by supervised MVA (OPLS-DA) performed considering separately treated and untreated samples (Figure 2a,b). Two models were obtained, both indicating mannitol as the principal discriminating metabolite, according to the sampling period. Increased levels of mannitol and lower levels of polyphenols (oleuropein and its derivatives) were observed during the early springtime sampling with respect to the early fall for both treated and untreated samples (S-line plots of Figure S2a,b). Nevertheless, the model parameters clearly indicated a major difference along the predictive component occurring for untreated with respect to treated samples (Q2 values 0.896 and 0.776 for the models of treated and untreated samples, respectively). This behavior is in agreement with a limiting effect of the treatment on the natural decrease of mannitol levels, occurring in the infected trees during the spring–summer season [22].

In order to better understand the reasons of the observed metabolic profiles general behavior, the cultivar related effects of the sampling periods and treatment were separately investigated for each of the *X. fastidiosa s*ubsp. *pauca* susceptible (Ogliarola salentina, Cellina di Nardò) and tolerant (Leccino) cultivars. Moreover, specific differences of Leccino vs. susceptible cultivars (considered as single class) were also studied.

### 2.3. Sampling Period Effects on Leaf Extract Metabolic Profiles for Treated and Untreated Naturally Infected Leccino, Cellina Di Nardò and Ogliarola Salentina

**Figure 3:**
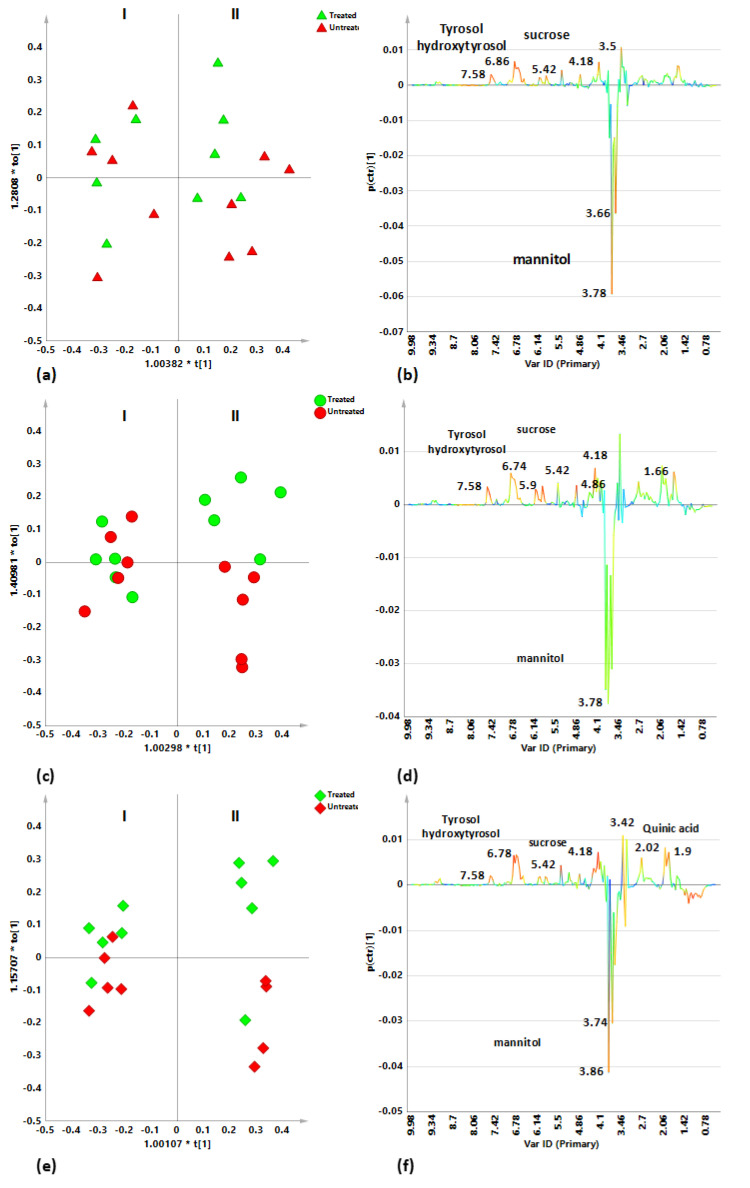
OPLS-DA t[1]/t[2] scores plot for infected Ogliarola salentina (triangle symbols) (**a**), Cellina di Nardò (circle symbols) (**c**) and Leccino (diamond symbols) (**e**), leaf samples related to I and II sampling periods. Red colored symbols indicated untreated sample. Green colored symbols indicated Dentamet^®^-treated samples. (**b**–**f**) S-line plots for Ogliarola salentina (**b**), Cellina di Nardò (**d**) and Leccino (**f**) related models visualizing the p(ctr)[1] loading colored according to the absolute value of the correlation loading, p(corr)[1].

Focusing on the two sampling, the metabolic profiles of the leaf extracts were analyzed separately, for each cultivar. In particular, a pairwise OPLS-DA analysis was performed considering only the two sampling classes (early springtime I, and early fall II) for both untreated and Dentamet^®^-treated samples. Although in this OPLS-DA model the treated and untreated samples were jointly considered and classified only according to the sampling period, a color code allows their identification also according to the treatment in the obtained scores plots (Figure 3). The supervised OPLS-DA analysis, performed on infected Ogliarola salentina leaf extracts samples, for the two sampling periods, resulted in a model where 1 + 1 + 0 components gave R2X = 0.567; R2Y = 0.874, Q2 = 0.698. A clear separation between the two sampling periods could be observed from the scores plot of the model (Figure 3a). The molecular components responsible for the observed separation were identified from the corresponding S-line plot (Figure 3b). Higher relative mannitol content and lower tyrosol, hydroxytyrosol and sucrose levels were observed for the first with respect to the second sampling. The two considered classes appeared quite scattered along the orthogonal component without a specific separation, between treated and untreated samples in the first sampling. Only a limited degree of differentiation between treated and untreated samples was observed in the second sampling (Figure 3a). Additionally, in the case of infected Cellina di Nardò leaf extract samples the pairwise OPLS-DA resulted in a model with good descriptive and predictive parameters (1 + 1 + 0 gave R2X = 0.488; R2Y = 0.924, Q2 = 0.813). Again, a clear separation between the two sampling periods was observed (Figure 3c). The S line plot for the model indicated, also in this case, higher levels of mannitol and lower tyrosol, hydroxytyrosol and sucrose content as discriminating for the first with respect to the second sampling (Figure 3d). Interestingly, although without a specific separation, between treated and untreated samples, the two considered classes appeared much less scattered (with respect to Ogliarola salentina) along the orthogonal component in the first sampling. On the other hand, higher level of samples dispersion and a clear discrimination between treated and untreated samples was observed along the orthogonal component, for the second sampling (Figure 3c). The OPLS-DA analysis for Leccino cultivar provided a good model described by 1 + 1 + 0 components giving R2X = 0.691, R2Y = 0.974 and Q2 = 0.958. A marked separation between the two sampling periods could be observed (Figure 3e). The S-line plot for the model reveals increased levels of mannitol among the discriminant metabolites for the first with respect to the second sampling. On the contrary, a higher relative content of tyrosol, hydroxytyrosol, sucrose, and quinic acid was observed for the second sampling (Figure 3f). The presence of high values of quinic acid, precursor in lignin biosynthesis [24], was already observed and reported in the literature [9]. Interestingly, along the orthogonal component, a compact group was obtained for the first sampling while a marked intraclass variability ascribable to a clear separation between treated and untreated samples, characterized the second sampling. Statistical parameters of supervised OPLS-DA models were summarized and reported in Table 2. The predictive ability of the models could be compared by evaluating their Q2 parameters values. The Q2 values observed for all the models described a satisfactory predictive ability indicating a well detectable discrimination between the two samplings for all the considered cultivars. However, according to the distinct Q2 values obtained for the OPLS-DA a clear rank (Leccino > Cellina di Nardò > Ogliarola salentina) for the metabolite profiles differentiation according to the sampling period could be observed for the three studied cultivars.

This cultivar dependence of the discrimination observed for the leaf extracts metabolic profiles, between the early springtime (I) and early fall (II) sampling of *X. fastidiosa* subsp. *pauca* infected trees, clearly suggests occurrence of different disease and season effects over the considered time span. Moreover, a further interesting cultivar dependence could be easily detected by observing the sample distribution along the first orthogonal component in the OPLS-DA models of Figure 3. Indeed, a different sample scattering characterizes the first with respect to the second sampling for each of the studied cultivar. Interestingly such a sample organization along the first orthogonal component appears to be related to the treatment occurrence or absence. As expected, although at a different extent for the three investigated cultivars, a marked cluster separation for the treated with respect to untreated samples arises after 6 months of treatment (early fall sampling). A specific analysis of treatment related effect on the metabolic profiles for the leaf extracts of *X. fastidiosa* subsp. *pauca* infected trees was therefore undertaken.

### 2.4. Cultivar-Related Treatment Effects for Naturally Infected Leccino, Cellina Di Nardò and Ogliarola Salentina over the Two Leaf Samplings

**Figure 4:**
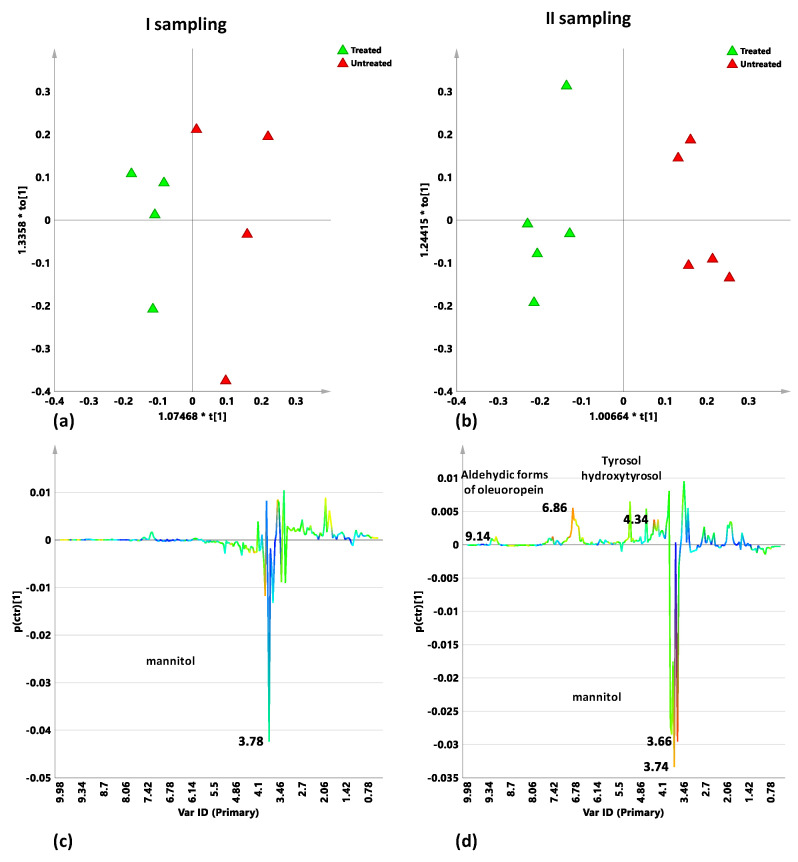
OPLS-DA t[1]/t[2] scores plot for infected control (red triangles) and Dentamet^®^-treated (green triangles) Ogliarola salentina leaf samples from I (**a**) and II (**b**) samplings. S-line plots for the I (**c**) and II (**d**) sampling related models visualizes the p(ctr)[1] loading colored according to the absolute value of the correlation loading, p(corr)[1].

In order to deeply analyze the treatment effects, specific pairwise comparison (treated vs. untreated) was then performed by supervised OPLS-DA analyses for each infected cultivar for the two different sampling times (early March, before the considered treatment and early October, after the considered treatment). The pairwise analysis, performed on the naturally infected Ogliarola salentina samples in the two sampling periods showed an increase of the separation between the two groups (naturally infected untreated and Dentamet^®^-treated samples) in the second with respect to the first sampling. In particular, for the first sampling, the analysis gave a model in which 1 + 1 + 0 components gave R2X = 0.499, R2Y = 0.803 and Q2 = −0.57 indicating absence of predictivity. Minimal separation between treated and untreated samples could be observed from visual inspection of the corresponding scores plot (Figure 4a). Nevertheless, the S line plot for the model indicated the presence of mannitol with higher relative content for the treated sample group (Figure 4c). In the second sampling the OPLS-DA analysis gave a better model with a good predictive parameter (1 + 1 + 0 components gave R2X = 0.468, R2Y = 0.947 and Q2 = 0.695), indicating a clearer separation between the two considered classes (treated and untreated naturally infected samples) (Figure 4b). The molecular components responsible for the separation were shown in the corresponding S line plot for the model (Figure 4d). Again, the presence of higher levels of mannitol for the Dentamet^®^-treated samples could be observed. On the other hand, untreated samples showed higher relative content of the disease biomarker quinic acid, tyrosol/hydroxytyrosol and aldehydic forms of oleuropein, as already observed and reported in previous work [15].

**Figure 5:**
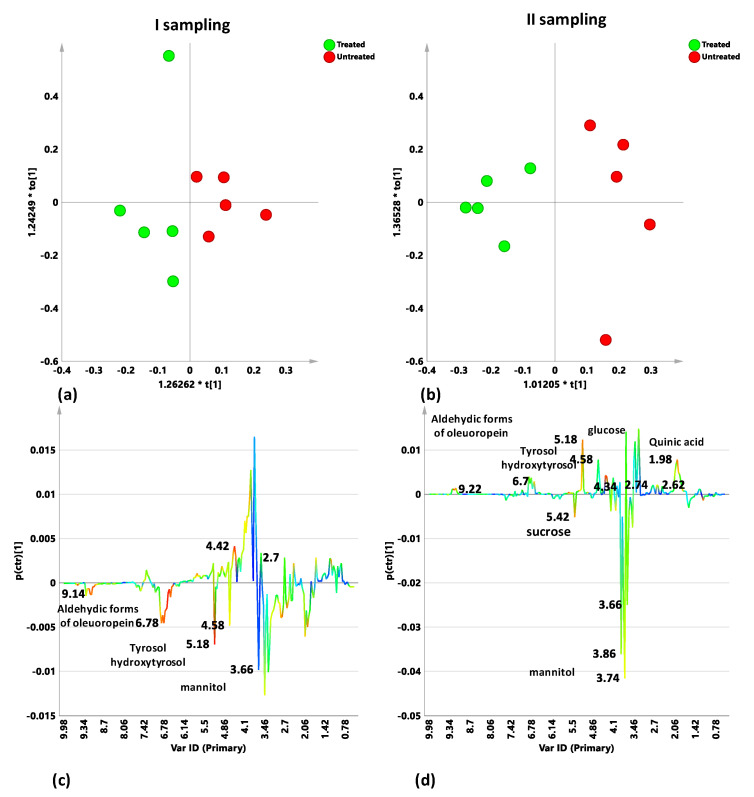
OPLS-DA t[1]/t[2] scores plot for infected control (red circles) and Dentamet^®^-treated (green circles) Cellina di Nardò leaf samples from I (**a**) and II (**b**) samplings. S-line plots for the I (**c**) and II (**d**) sampling related models visualizes the p(ctr)[1] loading colored according to the absolute value of the correlation loading, p(corr)[1].

The pairwise OPLS-DA analysis was therefore performed comparing treated and untreated naturally infected Cellina di Nardò leaf samples for each sampling period. For the first sampling a predictivity lacking model was obtained (1 + 1 + 0 components gave R2: 0.536; R2Y: 0.794 and Q2 = −1.01). The two classes represented by naturally infected treated and untreated Cellina di Nardò samples show minimal separation (Figure 5a). However, the S line plot for the model indicated the presence of higher level of mannitol and aldehydic forms of oleuropein for Dentamet^®^-treated samples (Figure 5c). The separation between the two classes improved by considering samples from the second sampling. The OPLS-DA model was described by 1 + 1 + 0 components giving R2X: 0.596, R2Y: 0.895 and Q2 = 0.763. According with the improved predictivity parameter Q2, a clearer separation between the two classes was observed (Figure 5b). The S line plot for the model indicated the presence of higher levels of mannitol and sucrose for Dentamet^®^-treated samples. The untreated samples, on the other hand, resulted characterized by higher content of quinic acid and aldehydic forms of oleuropein, together with tyrosol and hydroxytyrosol consistently with our previous findings [15] and recently reported literature data [25] (Figure 5d).

**Figure 6:**
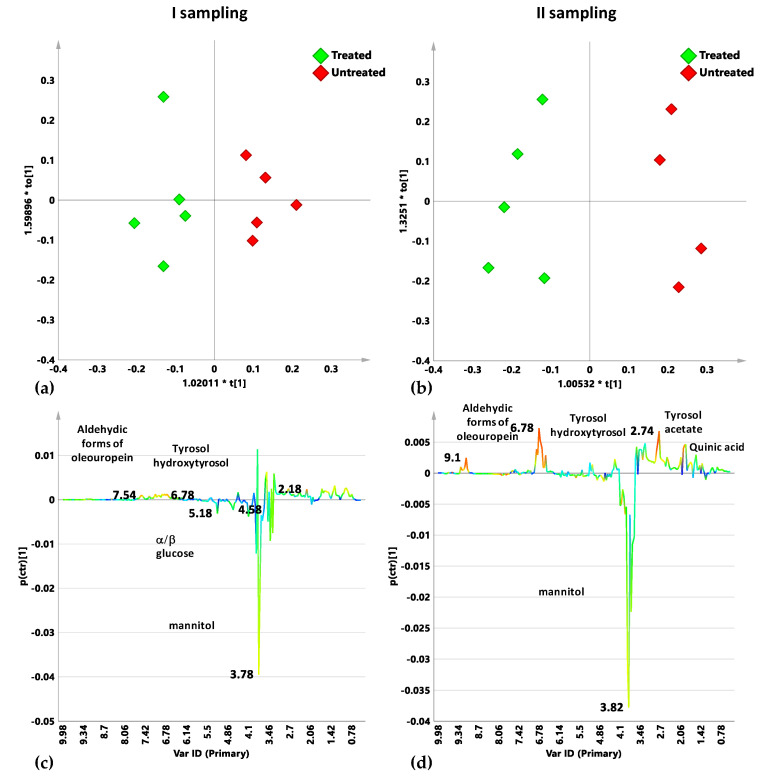
OPLS-DA t[1]/t[2] scores plot for infected control (red diamonds) and Dentamet^®^ treated (green diamonds) Leccino leaf samples from I (**a**) and II (**b**) samplings. S-line plots for the I (**c**) and II (**d**) sampling related models visualizes the p(ctr)[1] loading colored according to the absolute value of the correlation loading, p(corr)[1].

For the Leccino cultivar, the OPLS-DA pairwise model, for the first sampling, obtained with 1 + 1 + 0 components, gave R2X 0.39 and R2Y 0.887 as variation descriptive parameters and a very low but still positive predictive indicator Q2 0.323 (Figure 6a). The S-line plot for the model identified the variables responsible for differentiation between classes (Figure 6c). In particular, higher levels of secoiridoids such as oleuropein in its aldehydic forms and simple phenols such as tyrosol, hydroytyrosol characterized untreated infected Leccino samples. In turn, Dentamet^®^-treated samples showed higher values of mannitol and other monosaccharides (α/β glucose) than untreated. The OPLS-DA analysis for the samples collected during the second sampling showed a much better model differentiating untreated versus Dentamet^®^-treated infected samples since 1 + 1 + 0 components gave R2X = 0.563, R2Y = 0.945 and Q2 = 0.554 (Figure 6b). The S line plot for the model revealed that higher levels of phenolic compounds such as tyrosol and hydroxytyrosol were discriminating for untreated infected Leccino leaf samples (Figure 4d). On the other hand, Dentamet^®^-treated samples showed a higher content of mannitol with respect to untreated.

Statistical parameters of supervised OPLS-DA models were summarized and reported in Table 3. The predictive ability of the models which indicates the degree of class differentiation could be compared by evaluating their Q2 parameter values. For all the cultivars, an increase of the Q2 predictive parameter value could be observed in the second sampling. Moreover, a higher Q2 value indicates a higher discrimination between the considered classes. Thus, an increase in the separation between treated and untreated sample groups could be reported for all the cultivars. This could be ascribable to the 6 month treatment effect for the second sampling (October) in comparison with the first sampling (early March), performed before starting the first treatment of the considered year. Among cultivars, Cellina di Nardò and Ogliarola salentina showed a higher increase of Q2 parameters in the second sampling with respect to tolerant Leccino cultivar. Thus, in the case of Leccino cultivar, the Q2 parameter for OPLS-DA reveals a significant differentiation among treated and untreated samples also in the first sampling. This differentiation could be ascribable to a possible memory effect of the previous years’ treatment in Leccino cultivar not observed in the case of susceptible Ogliarola salentina and Cellina di Nardò cultivars. The presence of higher levels of mannitol as discriminating molecular component for the Dentamet^®^-treated samples was observed for all the studied cultivars in the two sampling, suggesting a general treatment related increase for this osmolyte, marker of hydric stress condition [25].

As known, among the olive trees’ morphological and physiological response to drought stress, the intracellular accumulation of mannitol, can be considered as a strategy to improve tolerance against water deficit thanks to its osmoprotectant and antioxidant ability to protect chloroplasts [26,27]. Moreover, as already reported [28], accumulation of mannitol was observed in olive leaves in response to foliar fertilization, suggesting an improvement of physiological performance and photosynthetic capability of olive trees. Interestingly, in the case of Cellina di Nardò samples, the observed higher content of sucrose in treated samples of the second sampling, also reported as a consequence of foliar fertilization in olive trees [28]. Moreover it should be noted that sucrose’s role as signaling molecule in response to cavitation was already described [10]. For the untreated samples, a higher relative content of phenolic compounds such as tyrosol and hydroxytyrosol moieties of oleuropein and its aldehydic forms were observed in the second sampling for all the analyzed cultivars, consistently with our previous findings [15] and recently reported literature data [25]. As already described in the literature, secoiridoids such as oleuropein and its derivatives are secondary metabolites, largely diffused in plants and with defense as the main role. Thus, the bitterness of many of this compounds has been considered to be a deterrent for herbivores [29]. The potential protective role exhibited by secoiridoids from *Olea europaea* L. for the prevention and treatment of cancers of inflammatory and reactive oxygen species (ROS)-mediated diseases was recently described in the literature [30,31]. Thus, the observed increase of secoiridoids content for the untreated infected cultivar could be also taken into account for possible biotechnological use in pharma industry. A higher content of quinic acid, a precursor in lignin biosynthesis [24], was observed for Cellina di Nardò and Leccino cultivars untreated samples, according to our previous findings [15]. The magnitude scale of the levels of discriminating metabolites for untreated vs. treated Ogliarola salentina, Cellina di Nardò, Leccino cultivars in the two sampling periods, given as Log2 fold change (FC) ratio of the mean of the buckets corresponding to selected distinctive unbiased NMR signals is reported in Figure S3.

### 2.5. Leccino vs. Susceptible Cultivars Differences in Infected Trees

In our previous studies, we studied the metabolic profiles of Ogliarola salentina and Cellina di Nardò, cultivars, very susceptible to *X. fastidiosa* subsp. *pauca* and we observed cultivar-specific polyphenols and sugar patterns [16]. Moreover, recently we have found the presence of a disease marker, namely quinic acid for both untreated Ogliarola salentina and Cellina di Nardò cultivars leaf samples [15]. Consequently, in order to deeply analyze and compare the metabolic response to both infection and treatment of tolerant Leccino with respect to susceptible cultivars we performed further pairwise supervised OPLS-DA analyses. In particular, the differences between Leccino class and the other cultivars (Ogliarola salentina and Cellina di Nardò), grouped as a single “susceptible class” were studied considering treatment occurrence and the two different sampling periods (I and II).

#### 2.5.1. Untreated Leccino vs. Susceptible Cultivars Trees: I and II Sampling

**Figure 7:**
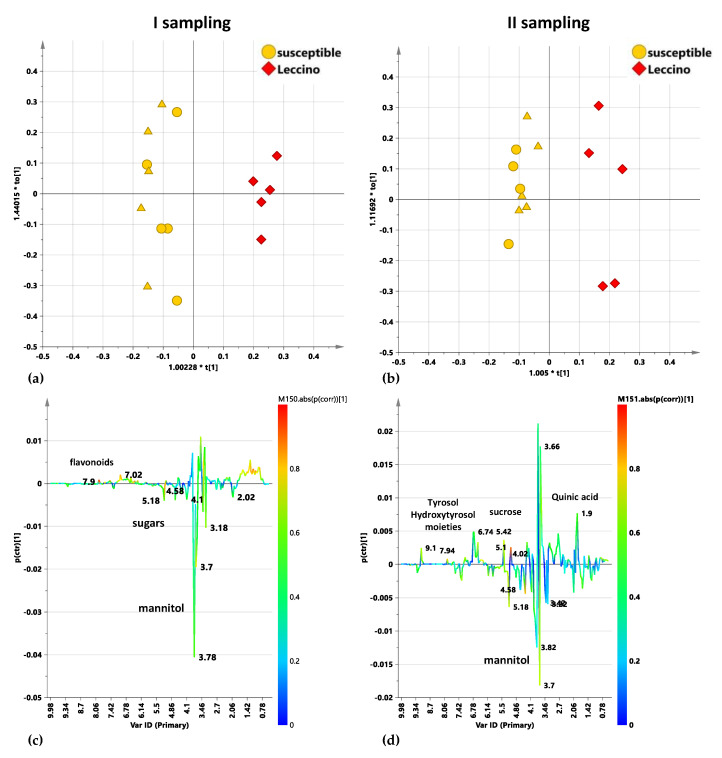
OPLS-DA t[1]/t[2] scores plot for Leccino and susceptible cultivar considering, infected control untreated leaf samples considering the first (**a**) and the second sampling (**b**). Different symbol style for Cellina di Nardò (circles) and Ogliarola salentina (triangles) susceptible cultivars are reported. S-line plots for the first (**c**) and the second sampling (**d**) related models visualizes the p(ctr)[1] loading colored according to the absolute value of the correlation loading, p(corr)[1]. Infected control trees.

In the first instance Untreated Leccino vs. susceptible cultivars trees, I and II sampling were considered. Spectral data analyzed by OPLS-DA showed a clear separation between untreated, infected Leccino with respect to susceptible cultivars in the first sampling (Figure 7a). The model was described by 1 + 1 + 0 components giving R2X = 0.668; R2Y = 0.953; Q2 = 0.811. The infection on the Leccino trees seemed to specifically modify the metabolic profiles with respect to the other here studied susceptible cultivars, with an increase of flavonoids, as observed from the S line plot for the model (Figure 4c). Higher amounts of flavonoids were already observed in infected Leccino compared to susceptible Cellina di Nardò plants [25,32]. On the contrary, in the untreated susceptible cultivars, variables ascribable to mannitol signals and indicating higher relative sugar content with respect to Leccino were observed. Generally, increased levels of sugars [15,16] and in particular mannitol [23] have been already reported for infected Ogliarola salentina and Cellina di Nardò cultivars. The clear separation of untreated susceptible samples with respect to Leccino resulted also from the second sampling OPLS-DA analysis, although with decreased values of descriptive and predictive parameters (1 + 1 + 0 components giving R2X = 0.645; R2Y = 0.948; Q2 = 0.684) (Figure 7b). The decrease of the model predictive index (Q2) could be related to a progression of the infection which smoothens the cultivar profile differences, as already reported in other work [15]. Beside the differences in mannitol and flavonoids content (already observed in the first sampling), the S line plot showed for the untreated Leccino samples with respect to susceptible cultivars also other variances consistent with a response to the disease progression. In particular, higher relative content of sucrose, hydroxytyrosol and tyrosol moieties as well as quinic acid were also observed. (Figure 7d). Interestingly, content of sucrose was already reported to be higher for Leccino with respect to other cultivars, and strictly related to the vegetative season [33]. The high constitutive sucrose content observed for Leccino has been also recently reported as involved in *X. fastidiosa* subsp. *pauca* resistance, being a signaling response molecule to cavitation [10]. Moreover, quinic acid, precursor in lignin biosynthesis [24], was already observed at higher values for Leccino with respect to susceptible cultivars [9].

#### 2.5.2. Dentamet^®^-Treated Leccino vs. Susceptible Cultivars Trees: I and II Sampling

**Figure 8:**
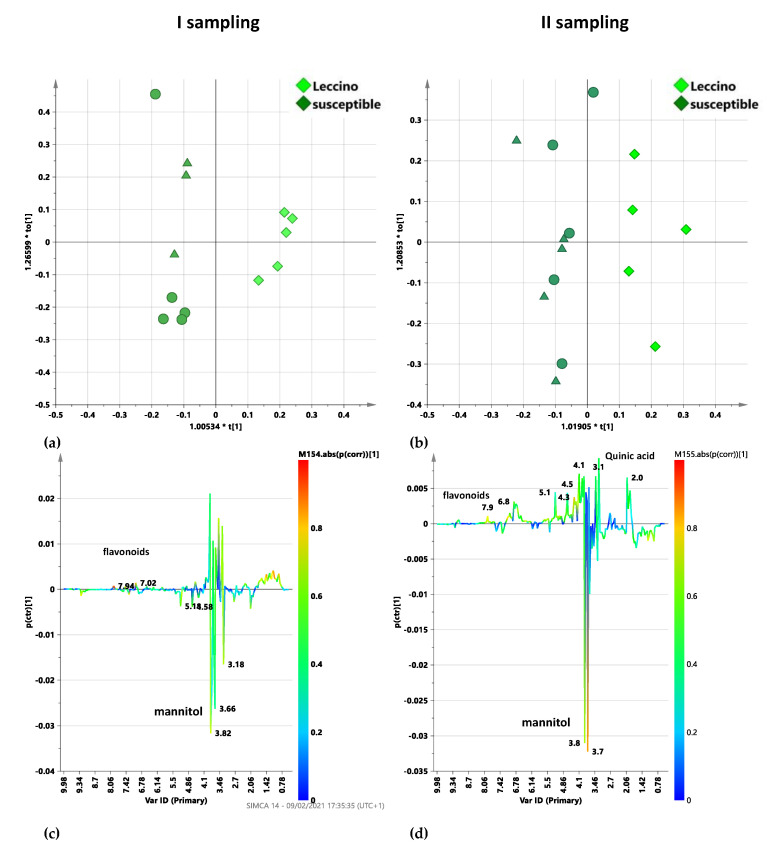
OPLS-DA t[1]/t[2] scores plot for infected Dentamet^®^-treated Leccino and susceptible cultivar leaf samples, considering the first (**a**) and the second (**b**) sampling. Different symbol styles for Cellina di Nardò (circles) and Ogliarola salentina (triangles) susceptible cultivars are reported. S-line plots for the, infected Dentamet^®^-treated considering the first (**c**) and the second (**d**) sampling related models visualizes the p(ctr)[1] loading colored according to the absolute value of the correlation loading, p(corr)[1].

In the first sampling, spectral data of Dentamet^®^-treated, infected trees, analyzed by OPLS-DA, showed again a clear separation between Leccino and the susceptible cultivars (Figure 8a). The model was described by 1 + 1 + 0 components giving R2X = 0.65; R2Y = 0.953; Q2 = 0.518. It should be noted that, being the first sampling arranged after 6 months suspension period and before the new treatment, the observed class separation could be only ascribable to a possible memory effect in Leccino cultivar. Interestingly, this feature was not observed in the case of susceptible Ogliarola salentina and Cellina di Nardò cultivars.

The S line plot for the model showed higher values for flavonoid molecules in Leccino than susceptible cultivar samples (Figure 8c). Moreover, susceptible cultivars (i.e., Ogliarola salentina and Cellina di Nardò) were characterized by higher relative content of mannitol. These results were similar to those observed for untreated samples comparison of Figure 7a. This suggests that for the Leccino, with respect to the susceptible cultivars, the previous cycle treatments’ effects are consistent with the natural specific response of the tolerant species against the disease progression. In the second sampling, the pairwise OPLS-DA analysis, performed on the infected Dentamet^®^-treated Leccino with respect to susceptible cultivars leaf samples, provided a model in which 1 + 1 + 0 components gave R2X = 0.696; R2Y = 0.827 and Q2 = 0.398 (Figure 8b). The relatively low prediction parameter (Q2) indicated a less marked separation between the two classes after the considered treatment. Therefore, the effect of 6 months of treatment seems to smooth the differences in the metabolic profiles of the two classes. The S line plot for the model (Figure 8d) showed a higher relative content of mannitol for the treated susceptible cultivars with respect to Leccino. On the contrary Leccino samples exhibited a higher content of quinic acid and flavonoids with respect to susceptible cultivars.

#### 2.5.3. Leccino vs. Susceptible Cultivars in Infected and Healthy Trees: General Remarks

Statistical parameters of supervised OPLS-DA models, showing Leccino vs. susceptible cultivars comparisons, related to untreated and treated trees for I and II sampling, are summarized and reported in Table 4. It should be noted that all the models resulted characterized by a good descriptive parameters R2X, R2Y. On the other hand, the predictive ability of the models which indicates the degree of class differentiation could be compared by evaluating their Q2 parameters values. Interestingly, for both untreated and treated cultivars a decrease of the Q2 predictive parameter value could be observed in the second sampling. In the untreated trees, these differences smoothing, occurring in the second sampling, may indicate the effect of the disease progression, which renders the metabolic profile of the infected Leccino leaves closer to that of susceptible cultivars. Nevertheless, this occurs by keeping along the 6 months observation the well-known differences of the infected Leccino with respect to susceptible cultivars (lower mannitol and higher sucrose, polyphenols and quinic levels) [9,10]. On the other hand, in the treated trees the differences reduction observed in the second sampling could be ascribed to the treatment effect which may result in a leveling response to the disease progression for susceptible cultivars with respect to Leccino. Note that also in this comparison, although reduced, some of the differences between infected Leccino and the susceptible cultivars (lower mannitol and higher quinic levels) remain along the 6 months observation. Interestingly, by focusing at the difference evaluation due to the treatment, in the 6 months observation of the overall multiyear therapy, higher levels of flavonoids and lower polyphenols, could be also reported in the comparison of Leccino with respect to susceptible cultivars.

**Figure 9:**
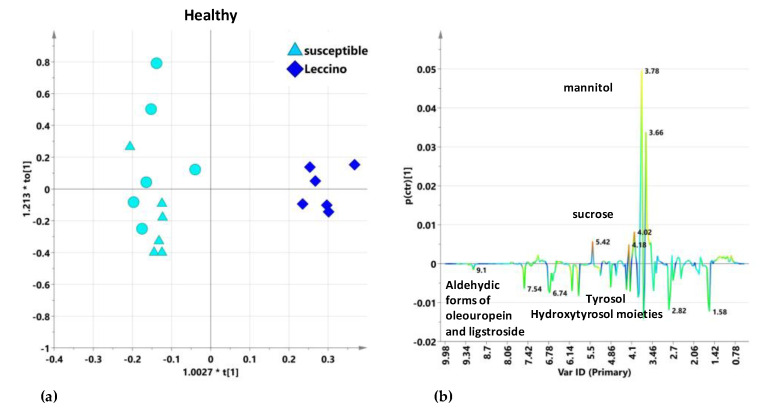
(**a**) OPLS-DA t[1]/t[2] scores plot for healthy Dentamet^®^-treated Leccino and susceptible cultivar leaf sample. Different symbol styles for Cellina di Nardò (circles) and Ogliarola salentina (triangles) susceptible cultivars are reported. (**b**) S-line plots for the model visualizing the p(ctr)[1] loading colored according to the absolute value of the correlation loading, p(corr)[1].

It should also be noted that the observed differences in the infected Leccino with respect to susceptible cultivars in untreated and treated trees, considered in the first sampling (before the start of the last treatment cycle), showed reversed behavior for mannitol levels with respect to the comparison of analogous classes of healthy trees. In this case, although the data refer to a different area officially declared free of infection, leaf extract ^1^H-NMR data of Leccino and susceptible cultivars healthy trees gave, in the OPLS-DA analysis, a quite stable model with 1 + 1 + 0 components and good descriptive and predictive parameters R2X = 0.729; R2Y = 0.959; Q2 = 0.85 (Figure 9a).

**Figure 10:**
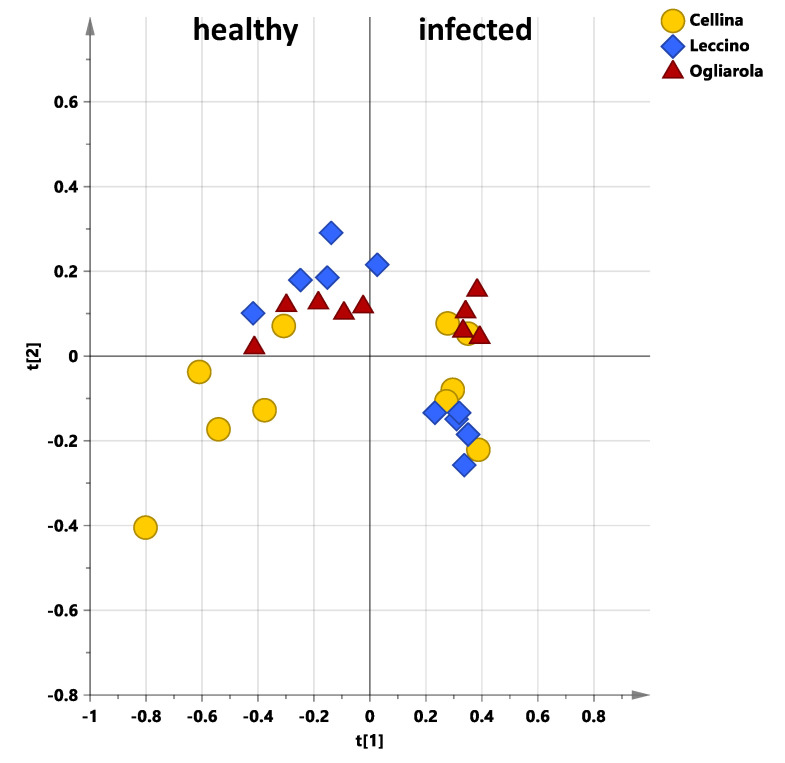
PCA t[1]/t[2] scores plot (three components gave R2X = 0.783 and Q2 = 0.631) for healthy and naturally infected Leccino, Cellina di Nardò and Ogliarola salentina leaf samples collected in the early springtime sampling. Healthy and naturally infected samples were collected in Grottaglie (Taranto province) and Galatone (Lecce Province), respectively. Different symbol styles for Leccino (diamonds), Cellina di Nardò (circles) and Ogliarola salentina (triangles) are reported.

As also observable from the model scores plot the two classes resulted well separated along the predictive component (class separation). Moreover, as expected [16], for the susceptible class a variation within the group was also seen along the orthogonal component. The related S-line plot allowed to identify the best discriminating variables (bucket related to NMR signals) between the two groups (Figure 9b). Indeed, Leccino healthy leaf samples were characterized by higher content of sucrose and mannitol with respect to the susceptible cultivars. The content of sucrose was already reported to be higher for Leccino with respect to other cultivar, and strictly related to the vegetative season [33]. The high constitutive sucrose content, observed for Leccino cultivar, could be also involved in the *X. fastidiosa* subsp. *pauca* resistance due to sucrose’s role as signaling molecule in response to cavitation [10]. At the same time, Leccino healthy leaf samples showed lower content of secoiridoids derivatives and phenyl alcohol moieties of oleuropein (tyrosol, hydroxytyrosol) with respect to the healthy susceptible cultivars, Ogliarola salentina and Cellina di Nardò. Previous transcriptomics studies results also indicated that *X. fastidiosa* subsp. *pauca* is perceived by olive trees of both tolerant (Leccino) and susceptible (Ogliarola salentina) cultivars, in which it perturbs differently gene expression [8]. In particular, analysis of these differentially expressed genes showed a differential response strongly involving the cell wall [8]. On the other hand, although the origin of samples in two different geographical areas prevented proper magnitude scale levels of discriminating metabolites analysis, the observed differences for all the here considered cultivars appeared much more enhanced for healthy with respect to infected trees. This result, which could be clearly observed even in a simple PCA model (Figure 10) is in accordance with previously reported data [16] that first suggested the use of ^1^H-NMR profiling for disease diagnosis and treatment effects follow up.

## 3. Materials and Methods

### 3.1. Sampling

Samplings were carried out in olive orchards planted with Leccino, Ogliarola salentina or Cellina di Nardò cultivars. Trees infected by *X. fastidiosa* subsp. *pauca* were located in a farm of Galatone (province of Lecce, Apulia, Italy) [21]. Healthy trees of the same cultivars growing in Grottaglie (Taranto province, Apulia, Italy) in an area officially declared free of *X. fastidiosa* subsp. *pauca*, according to the regional phytosanitary service of Apulia, were also sampled. In particular, a total of 15 leaf samples (five samples for each cultivar) from Cellina di Nardò, Ogliarola salentina and Leccino healthy trees were collected in early March 2019. The occurrence or absence of *X. fastidiosa* was molecularly ascertained [34] in each olive orchard. Trees had an age ranging from 60 to 80 years and were trained according to the traditional agronomical practices of Salento area (open vase, large space between the trees, no irrigation). In the olive grove of Galatone, a plot of Leccino, Ogliarola salentina and Cellina di Nardò trees was sprayed on the canopy with Dentamet^®^, a mixture of zinc and copper complex with hydracids of citric acid, already known as CE approved foliar fertilizer and specifically tested against *X. fastidiosa* as described in the literature [16]. To the best of our knowledge there are no other field treatments whose effectiveness was validated by PCR or metabolomics analysis [35]. The spray treatments started in 2017, at a dose of 3.9 L per hectare, once per month, starting from early April to early September, by using an atomizer [20]. Untreated infected olive trees of the same cultivars growing in the bordering groves were also sampled as controls. In 2019, a total of 60 leaf samples were collected in two sampling times (early March, 6 months after the last treatment and before the first treatment and early October after the last treatment of the year) as follows: 30 Dentamet^®^-treated Leccino, Ogliarola salentina and Cellina di Nardò leaf samples (10 samples for each cultivar) taken at Galatone olive grove and 30 Leccino, Ogliarola salentina and Cellina di Nardò leaf samples (10 samples for each cultivar) taken at Galatone olive groves bordering the Dentamet^®^-treated one. The leaf samples were put in plastic bags into a refrigerated box and taken to the laboratory for the analyses. Symptom progression over the sampling period was also molecularly (PCR) and visually assessed and the results are reported elsewhere [21].

### 3.2. Sample Preparation for ^1^H-NMR Analysis 

Samples were prepared according to the experimental procedure as reported in literature [14]. Briefly, olive leaf samples (each one containing 20 leaves) were plunged into liquid N_2_ and ground to a fine powder with a stainless-steel blender. Ground leaves were transferred into a plastic tube and placed in a freeze dryer for 48 h. Lyophilized plant material (100 mg) was weighted into an autoclaved 2 mL Eppendorf tube. Thereafter, 0.75 mL of CD_3_OD and 0.75 mL of KH_2_PO_4_ buffer in D_2_O (pH 5.9) containing 0.05% *w*/*v* TSP-*d*4 (sodium salt of trimethylsilylpropionic acid) were added to each sample. The content of the Eppendorf tubes was mixed thoroughly with a vortex mixer at room temperature for 1 min and then sonicated for 10 min at room temperature. Samples were spun down in a microcentrifuge at 17,000× *g* for 20 min; then, 700 µL of the supernatant were filled into a 5 mm NMR tube.

### 3.3. H-NMR Spectra Acquisition and Processing

All spectra were acquired at a constant temperature (300 K) on a Bruker Avance III 600 MHz Ascend NMR Spectrometer (Bruker Italia, Milano, Italy), operating at 600.13 MHz, equipped with a TCI cryoprobe (inverse Triple Resonance Cryoprobe Prodigy), incorporating a z axis gradient coil and automatic tuning-matching (ATM). Experiments were acquired in automation mode after loading individual samples on an integrated Bruker Automatic Sample Changer, interfaced with IconNMR software (Bruker). For each sample a ^1^H-NMR spectrum was acquired with water signal suppression (Bruker pulseprogram zgcppr), in a spectral window of 20.0276 ppm (12,019.230 Hz), 64 scans and a 90° pulse of 7.620 µsec. After the acquisition, the standard FID processing procedures were carried out, by using TopSpin 3.5 (Bruker, Biospin, Italy), such as the Fourier transform (mathematical operation that converts signals into a frequency spectrum), the phase and baseline correction, and 0.3 Hz line broadening. All the NMR spectra were calibrated with respect to the internal standard TSP (δ = 0.00 ppm). The characterization of the metabolites was determined by the analysis of two-dimensional homo- and heteronuclear NMR spectra (2D ^1^H J-resolved, ^1^H COSY, ^1^H-^13^C HSQC and HMBC) and by comparison with the literature data. The NMR spectra were converted to a suitable form for multivariate analysis by Amix 3.9.15 (Analysis of Mixture, Bruker BioSpin GmbH, Rheinstetten, Germany) software. Specifically, each NMR spectrum was segmented, into areas or histograms, with a fixed base width of 0.04 ppm (“normal rectangular bucketing”). The bucket tables thus obtained were subjected to a standardization procedure, in order to minimize the possible differences in concentration of the various metabolites due to sample preparation and/or acquisition conditions. Subsequently, the data matrices (buckets) were subjected to centering and scaling operations: the Pareto scaling method, obtained by dividing each variable by the square root of the variable standard deviation centered around the mean value, was applied [36]. The total sum normalization was applied to minimize small differences due to metabolites concentration and/or experimental conditions among samples [36,37]. The data table, generated by all aligned buckets row reduced spectra, was used for further multivariate data analysis. Each bucket row represents the entire NMR spectrum, with all the molecules in the sample. Moreover, each bucket, in a buckets row reduced spectrum, is labeled with the value of the central chemical shift for its specific 0.04 ppm width. The variables used as descriptors for each sample in chemometric analyses are the buckets.

### 3.4. Multivariate Statistical Analysis

After the data processing step, an exploratory and discriminating analysis was performed, using a multivariate statistical approach (MVA), with the help of the Simca-P version 14 (Sartorius Stedim Biotech, Umeå, Sweden) software. In particular, the principal components analysis (PCA) and the orthogonal projections to latent structures discriminant analysis (OPLS-DA) were performed. The PCA is used in the first data processing step, in order to obtain a general description of the samples’ distribution and their possible grouping in homogeneous clusters [38]. PCA is aimed at extracting the maximum possible information from a multivariate data structure, summarizing it in a few linear combinations of the variables themselves [39]. The assessment of the correlation between the cluster distribution of the analyzed samples (observed by PCA) and the considered classes is therefore carried out by using supervised multivariate statistical analyses such as OPLS-DA (orthogonal projections to latent structures discriminant analysis, OPLS-DA). The OPLS-DA is a modification of the PLS-DA method which filters out variation not directly related to the focused discriminating response and is suited for highlighting class discriminating variable in the two class problems [40,41]. The result is a model with improved interpretability. The validity and the degree of over fit for statistical models were checked by using internal cross-validation default method (7-fold) and with permutation test (40 permutations) [42]. The R2 and Q2 parameters were used to describe the quality of the model. The first (R2) is a cross validation parameter defined as the explained variance of the models and indicates goodness of fit. The second (Q2) represents the portion of variance in the data predictable by the model [43]. The variables responsible for the observed discrimination were identified by using the statistical tool S-line plot. The S-line plot is tailor-made for NMR spectroscopy data and creates a plot of the loading vectors for the first two components, loading colored according to the absolute value of the correlation scaled loading, p(corr)[1] [42]. Analysis of variance (one way-ANOVA) with Tukey’s honestly significant difference (HSD) post hoc test were used to examine mean differences among discriminating metabolite content for studied cultivars. Statistical significance was set at least at an adjusted *p*-values < 0.05

## 4. Conclusions

In the present study, we evaluated by ^1^H-NMR and MVA the metabolic profiles of the *X. fastidiosa* subsp. *pauca*-tolerant cultivar Leccino in comparison with the susceptible cultivars Ogliarola salentina and Cellina di Nardò either upon natural infection by the bacterium or after the treatments with Dentamet^®^. A specific timespan (6 months treatment April–September after 6 months suspension) was chosen during a multiyear on field usage schedule. The metabolic profiles of each cultivar were analyzed focusing on the two samplings (March, before the start and October after the end of treatments). Unsupervised and supervised MVA models, obtained for untreated and treated samples, respectively, demonstrated a different degree of separation along the first component. This separation, more pronounced in untreated with respect to treated samples, was found essentially related to the mannitol contribution. An interesting limiting effect of the treatment on the natural decrease of mannitol content, during the spring–summer seasons was observed. The analysis of the specific influence of sampling period on metabolic profiles for infected Leccino, Cellina di Nardò and Ogliarola salentina indicated a specific rank (Leccino > Cellina di Nardò > Ogliarola salentina) for the observed discrimination between the two sampling periods. Moreover, an increasing intraclass discrimination (treated vs. untreated), specific for the second sampling (Leccino > Cellina di Nardò > Ogliarola salentina), was also observed. This suggested the occurrence of different levels of disease, season and treatment effects over the considered time span for the considered cultivars. Specific MVA analyses of treatment-related effects gave a relevant increase of the Q2 predictive model parameters indicating a higher discrimination in the second sampling for treated vs. untreated trees. This result ascribable to the 6 months treatment with the biocomplex resulted much more enhanced for Cellina di Nardò and Ogliarola salentina with respect to tolerant Leccino. On the other hand, in the first sampling, a significant differentiation among treated and untreated samples was only observed for Leccino, suggesting a possible memory effect of previous years treatment specific for this cultivar. Comparison of tolerant Leccino with respect to the other susceptible cultivars (Ogliarola salentina and Cellina di Nardò), grouped as a single class, demonstrated a general (treated and untreated) smoothing of the observed differences in the second with respect to the first sampling. In the untreated trees, this effect could be ascribed to the disease progression, which renders the infected Leccino metabolic profile closer to that of susceptible cultivars. On the other hand, a similar effect could be observed in the treated trees, due to a possible treatment-related leveling response to the disease progression, enhanced for susceptible cultivars with respect to Leccino. Finally, the observed differences in the infected Leccino with respect to susceptible cultivars showed interesting reversed behavior for mannitol levels with respect to the analogous comparison for healthy trees. The present study clearly demonstrates the effectiveness of ^1^H-NMR-based metabolic profiling and MVA for the evaluation of specific metabolic responses of the *X. fastidiosa* subsp. *pauca*-infected tolerant Leccino cultivar compared to the susceptible cultivars. Noteworthy, the obtained information was also related to seasonal related sampling periods and Dentamet^®^ treatments during a multiyear on field usage schedule. The overall results could be useful for better understanding the plant defensive mechanisms and planning possible improved curative actions against *X. fastidiosa* subsp. *pauca* infection.

## Figures and Tables

**Table 1:** Summary of the experimental trials with Dentamet^®^ treatments (T) and leaf sampling (S) periods over 2017, 2018 and 2019.

	J	F	M	A	M	J	J	A	S	O	N	D
2017				T	T	T	T	T	T			
2018				T	T	T	T	T	T			
2019			S	T	T	T	T	T	T	S		

**Table 2:** Statistical parameters of supervised OPLS-DA models for first (I) and second (II) sampling periods in the naturally infected Leccino, Ogliarola salentina and Cellina di Nardò cultivars. R2X and R2Y indicate the fraction of variance of the X and Y matrix, respectively. Q2 is a goodness of prediction parameter representing the portion of variance in the data predictable by the model.

I vs. II Sampling OPLS-DA (1 + 1 + 0)	R2X	R2Y	Q2
Ogliarola salentina	0.567	0874	0.698
Cellina di Nardò	0.488	0.924	0.813
Leccino	0.691	0.974	0.958

**Table 3:** Statistical parameters of supervised OPLS-DA models for untreated and treated naturally infected Leccino, Ogliarola salentina and Cellina di Nardò cultivars. R2X and R2Y indicate the fraction of variance of the X and Y matrix, respectively. Q2 is a goodness of prediction parameter representing the portion of variance in the data predictable by the model.

Untreated vs. Treated OPLS-DA (1 + 1 + 0)	Sampling	R2X	R2Y	Q2
Ogliarola salentina	I	0.489	0.803	−0.57
	II	0.465	0.947	0.695
Cellina di Nardò	I	0.536	0.704	−1.01
	II	0.596	0.895	0.763
Leccino	I	0.39	0.887	0.323
	II	0.563	0.945	0.554

**Table 4:** Statistical parameters of supervised OPLS-DA models for first (I) and second (II) sampling periods in the treated and untreated naturally infected Leccino and susceptible cultivars. R2X and R2Y indicate the fraction of variance of the X and Y matrix, respectively. Q2 is a goodness of prediction parameter representing the portion of variance in the data predictable by the model.

Leccino vs. Susceptible Cultivars OPLS-DA (1 + 1 + 0)	Sampling	R2X	R2Y	Q2
Untreated samples	I	0.668	0.953	0.811
	II	0.645	0.948	0.684
Treated samples	I	0.65	0.953	0.518
	II	0.696	0.827	0.398

## Data Availability

Data is contained within the article and in the supplementary material.

## Supplementary Materials

The following are available online at https://www.mdpi.com/2223-7747/10/4/772/s1, Figure S1: PCA scores plot for untreated (a) and treated (b) naturally infected Leccino, Cellina di Nardò and Ogliarola salentina leaf samples comparing the I and II sampling periods. Line plot for the untreated (c) and treated (d) related models, indicating the ^1^H-NMR chemical shifts of the signals, characteristic of specific metabolites, discriminating the classes along t[1] and colored according to the correlation-scaled loading (* p(corr) ≥ |0.5|), Figure S2. S-Line plots for Figure 2 OPLS–DA models. Untreated (a) and treated (b) naturally infected Leccino, Cellina di Nardò and Ogliarola Salentina leaf samples comparing the I and II sampling periods, visualizing the p(ctr)[1] loading colored according to the absolute value of the correlation loading, p(corr)[1]. Figure S3. Magnitude scale of the levels of discriminating metabolites comparison between untreated and treated in the two sampling periods Ogliarola salentina ((a) and (b)), Cellina di Nardò ((c) and (d)), Leccino ((e) and (f)) cultivars provided as values of −Log2 (FC). Metabolites with −Log2 (FC) negative values have higher concentration in untreated samples, while −Log2 (FC) positive values indicated metabolites with higher concentration treated samples. Statistical significance (one-way analysis of variance (ANOVA)) was set at least at an adjusted *p*-values < 0.05 and indicated with 0 ‘***’ 0.001 ‘**’ 0.01 ‘*’ 0.05 ‘·’.
