# Supplementary material for: Olive Cultivars Susceptible or Tolerant to Xylella fastidiosa Subsp. pauca Exhibit Mid-Term Different Metabolomes upon Natural Infection or a Curative Treatment
Source: Plants (Basel). 2021 Apr 15;10(4):772. doi: 10.3390/plants10040772 (PMC8103516; doi:10.3390/plants10040772)
Supplement: Supplementary file 1 [file plants-10-00772-s001.pdf]

plants

MDPI

Supplementary material

Olive cultivars susceptible or tolerant to *Xylella fastidiosa* subsp. *pauca* exhibit medium-term different metabolomes upon natural infection or a curative treatment

Chiara Roberta Girelli [1], Laura del Coco [1], Federica Angilè [1], Marco Scortichini [2] and Francesco Paolo Fanizzi [1,*]

[1] Department of Biological and Environmental Sciences and Technologies, University of Salento, Prov.le Lecce-Monteroni, 73100 Lecce, Italy; chiara.girelli@unisalento.it (C.R.G.); federica.angile@unisalento.it (F.A.); laura.delcoco@unisalento.it (L.D.C.); marco.scortichinifp.fanizzi@unisalento.it (F.P.F.)
[2] Council for Research in Agriculture and Economics – Research Centre for Olive, Fruit and Citrus Crops, Via di Fioranello, 52; I-00134 Roma, Italy; marco.scortichini@crea.gov.it (M.S.)
* Correspondence: fp.fanizzi@unisalento.it; Tel.: +39-0832-29265)

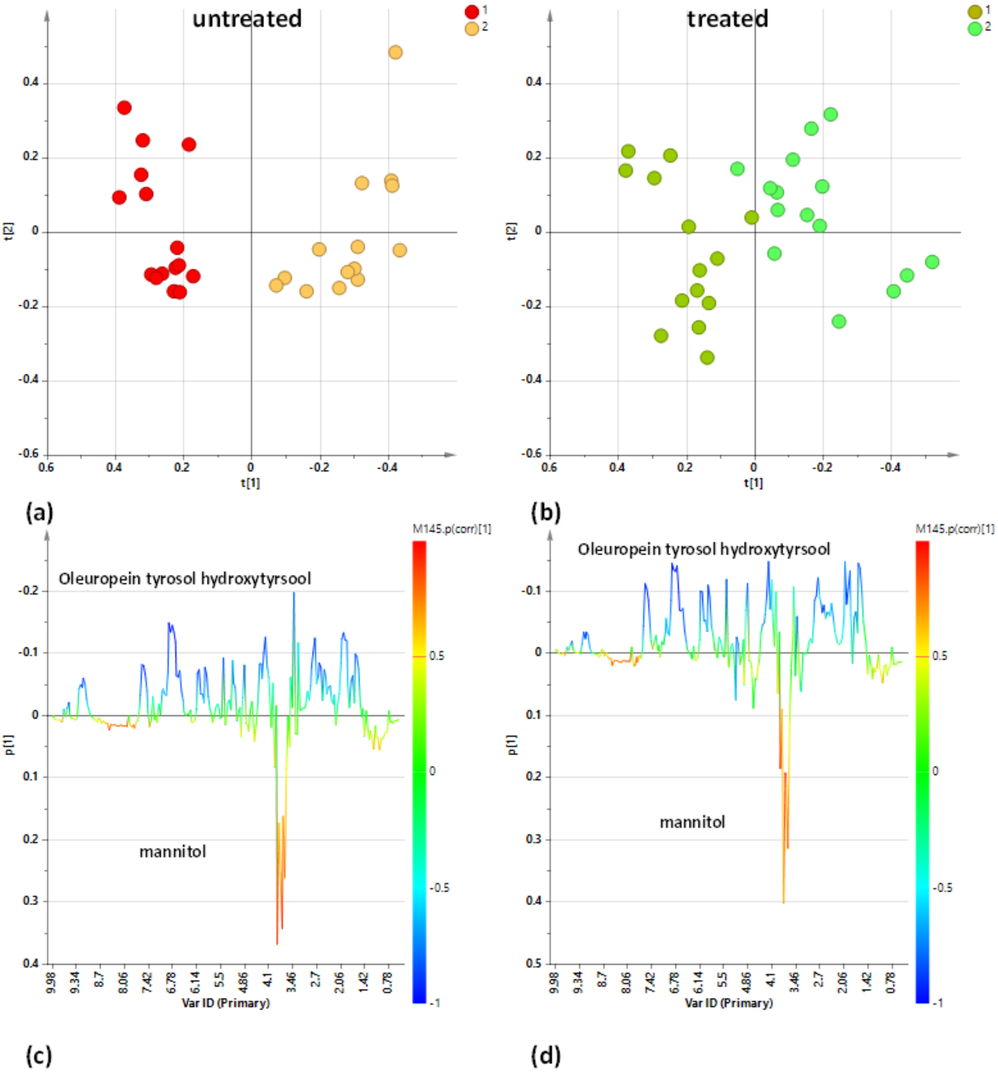

Figure S1. PCA scores plot for untreated (**a**) and treated (**b**) naturally infected Leccino, Cellina di Nardò and Ogliarola salentina leaf samples comparing the I and II sampling periods. Line plot for the untreated (**c**) and treated (**d**) related models, indicating the ^{1}H NMR chemical shifts of the signals, characteristic of specific metabolites, discriminating the classes along t[1] and coloured according to the correlation-scaled loading (p(corr) ≥|0.5|).

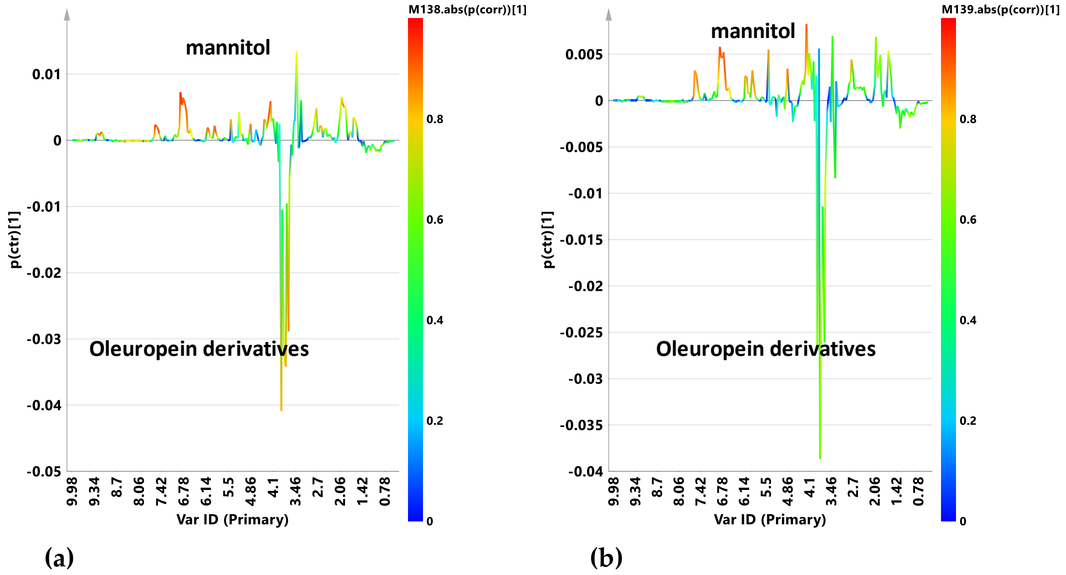

Figure S2. S-Line plots for Figure 2 OPLS–DA models. Untreated (**a**) and treated (**b**) naturally infected Leccino, Cellina di Nardò and Ogliarola Salentina leaf samples comparing the I and II sampling periods, visualizing the p(ctr)[1] loading colored according to the absolute value of the correlation loading, p(corr)[1].

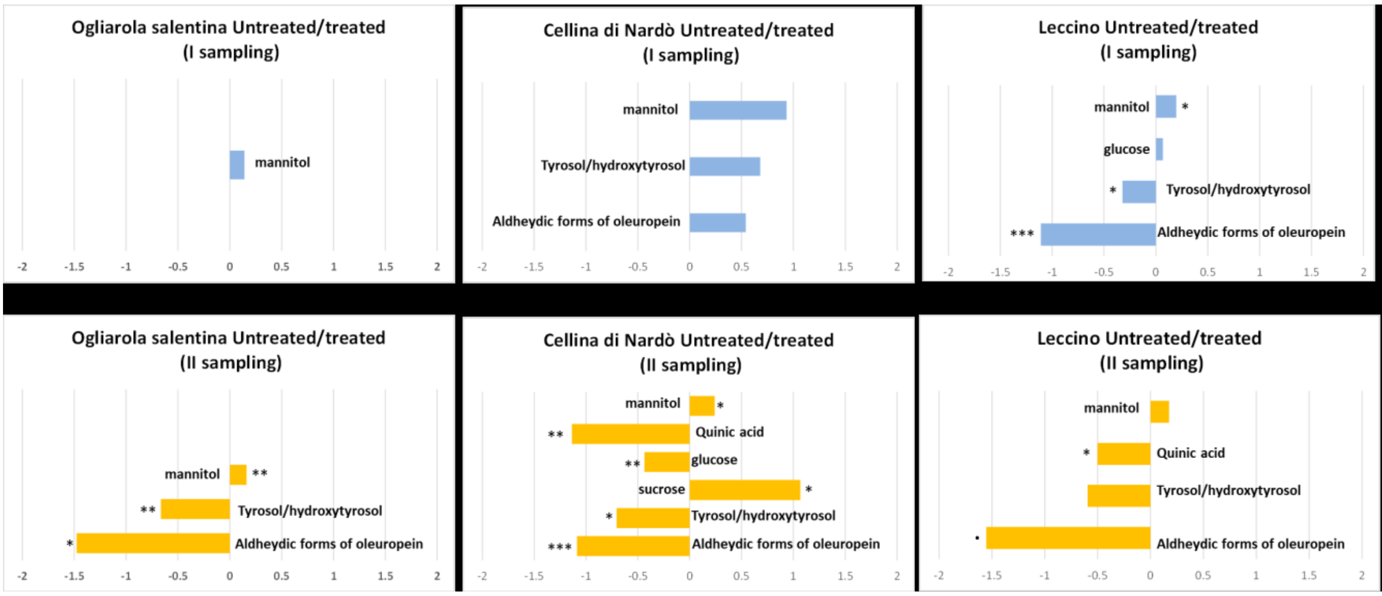

Figure S3. Magnitude scale of the levels of discriminating metabolites comparison between untreated and treated in the two sampling periods Ogliarola salentina ((a) and (b)), Cellina di Nardò ((c) and (d)), Leccino ((e) and (f)) cultivars provided as values of − Log2 (FC). Metabolites with − Log2 (FC) negative values have higher concentration in untreated samples, while − Log2 (FC) positive values indicated metabolites with higher concentration treated samples. Statistical significance (One-way analysis of variance (ANOVA) was set at least at an adjusted p-values < 0.05 and indicated with 0 ‘***’ 0.001 ‘**’0.01 ‘*’ '0.05 ' ·'.
